# Supplementary material for: Comparative Proteomics and Genome-Wide Druggability Analyses Prioritized Promising Therapeutic Targets against Drug-Resistant Leishmania tropica
Source: Microorganisms. 2023 Jan 16;11(1):228. doi: 10.3390/microorganisms11010228 (PMC9860978; doi:10.3390/microorganisms11010228)

Figure S1: Docking results of top ten hit molecules in the Pharmit resource showing 3D images of the molecular interaction of compounds C1-C10 with the LtUGPase.

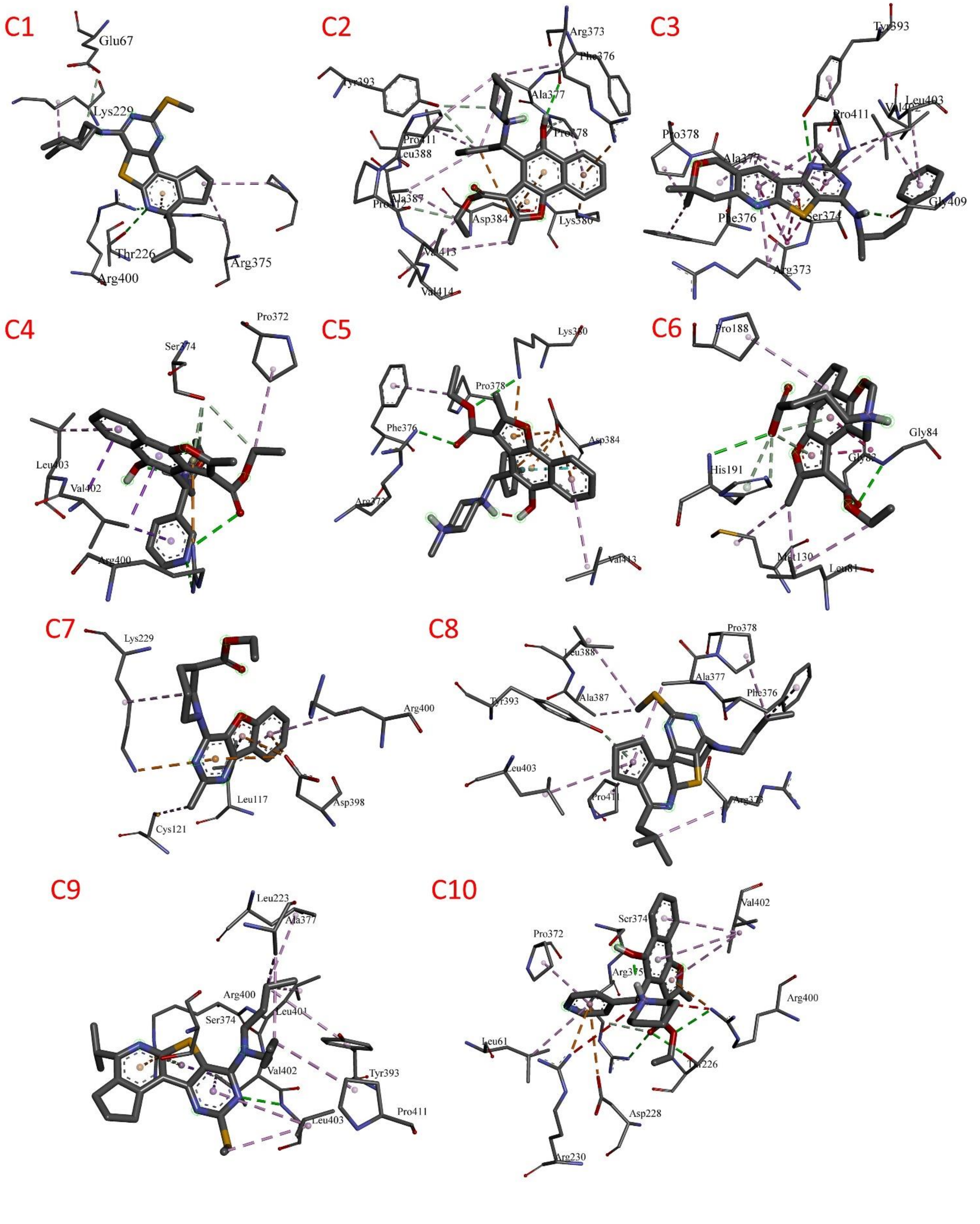

Figure S2: Docking results of top ten hit molecules in the Pharmit resource showing 2D images of the molecular interaction of compounds C1-C10 with the LtUGPase.

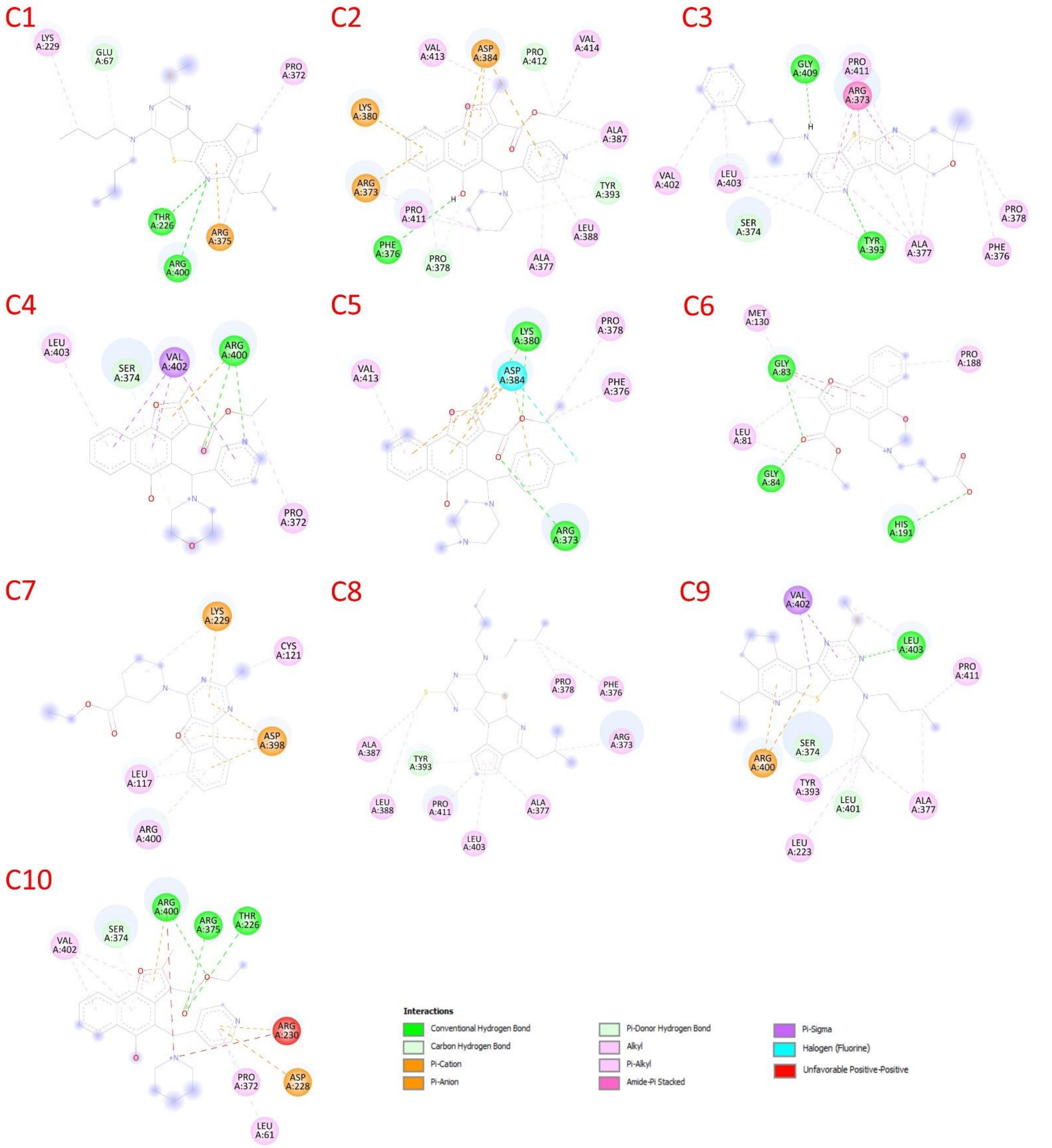

Figure S3: Docking results of top ten hit molecules in the Pharmit resource showing 3D images of the molecular interaction of compounds C1-C10 with the LtPCNA.

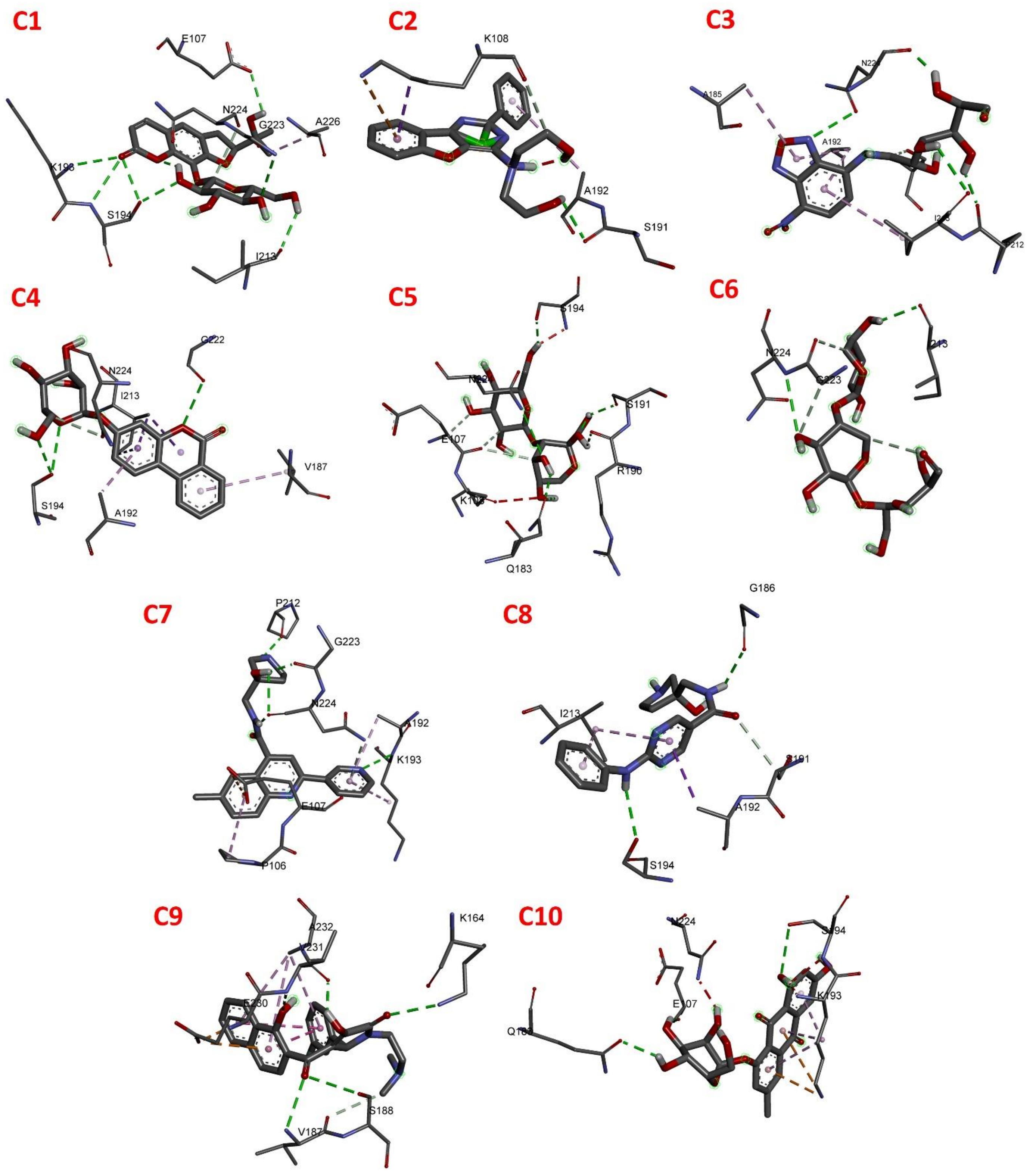

Figure S4: Docking results of top ten hit molecules in the Pharmit resource showing 2D images of the molecular interaction of compounds C1-C10 with the LtPCNA.

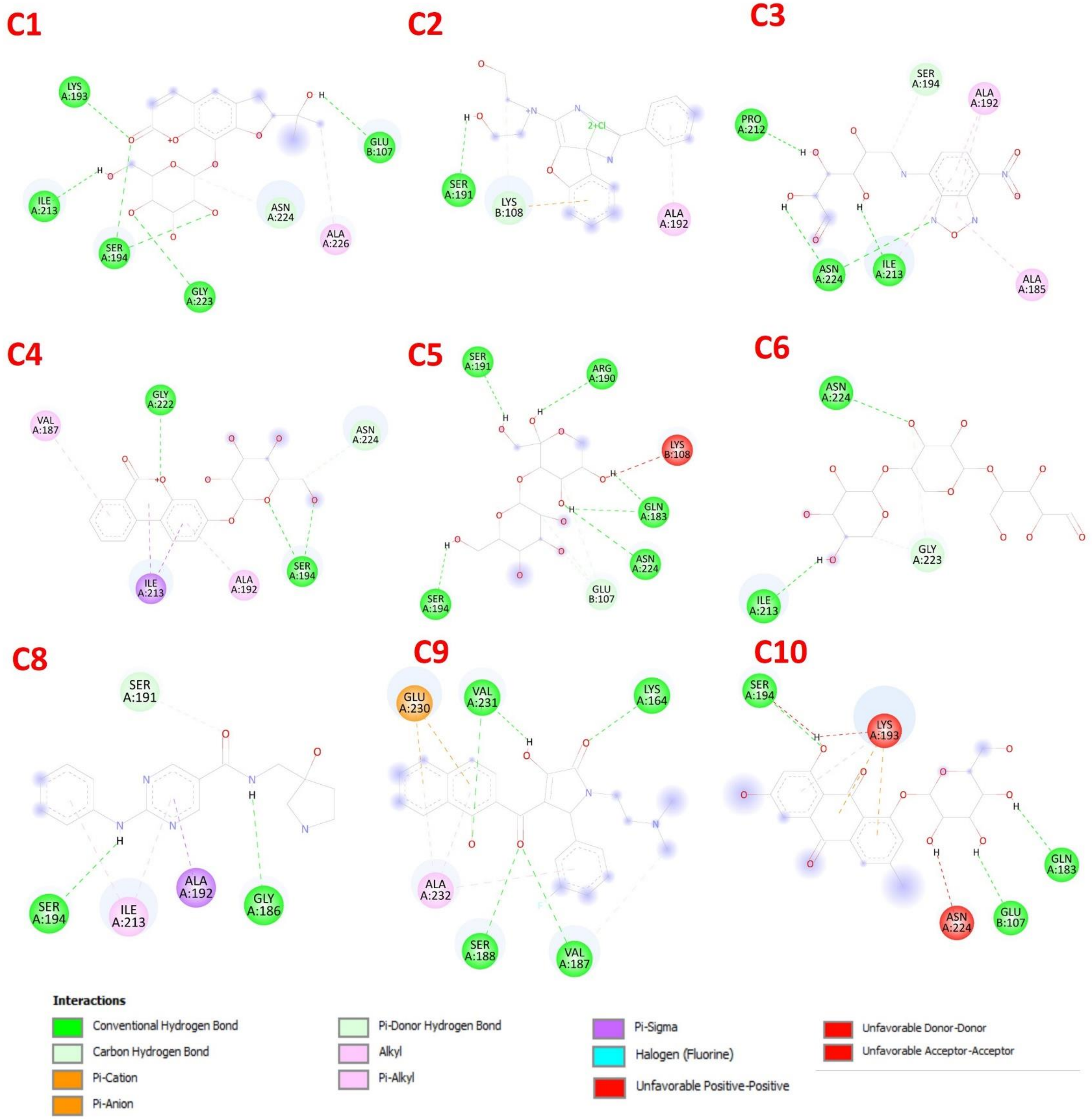

Supplement: Supplementary file 1 [file microorganisms-11-00228-s001.zip › microorganisms-2116659-supplementary/Supplementary_Figures.pdf]
