# Supplementary material for: Comparative Proteomics and Genome-Wide Druggability Analyses Prioritized Promising Therapeutic Targets against Drug-Resistant Leishmania tropica
Source: Microorganisms. 2023 Jan 16;11(1):228. doi: 10.3390/microorganisms11010228 (PMC9860978; doi:10.3390/microorganisms11010228)
Supplement: Supplementary file 1 [file microorganisms-11-00228-s001.zip › microorganisms-2116659-supplementary/Supplementary_Tables.pdf]

Table S1: The prediction of ADME parameters, pharmacokinetic properties, druglike nature, and medicinal chemistry friendliness of top 10 lead compounds predicted potent inhibitors against UGPase target.

**Serial No.**	**Compounds (MolPort IDs)**	**H-Bond Acceptors**	**H-Bond Donors**	**TPSA (Å2)**	**Consensus Log $P_{o/w}$**	**Molar Refractivity**	**GI Absorption**	**Log S**	**BBB Permeant**	**P-gp Substrate**	**Log Kp (skin permeation) cm/s**	**Drug likeness based on Lipinski rule**	**Bioavailability Score**	**PAINS (alert)**	**Brenk (alert)**	**Synthetic accessibility**
**C1**	MolPort-002-619-190	3	0	95.45	6.74	139.50	low	-7.48 Poorly soluble	No	Yes	-3.28 cm/s	Yes; 1 violation: MLOGP>4.15	0.55	0 alert	0 alert	4.17
**C2**	MolPort-000-451-699	6	1	75.8	4.47	132.78	High	-5.87 Moderately soluble	No	Yes	-5.36 cm/s	Yes; 0 violation	0.55	0 alert	1 alert: mannich_A	4.29
**C3**	MolPort-000-730-162	4	1	88.17	5.3	129.26	High	-6.24 Poorly soluble	No	Yes	-4.88 cm/s	Yes; 0 violation	0.55	0 alert	0 alert	4.33
**C4**	MolPort-000-451-697	7	1	85.03	3.63	129.05	High	-5.11 Poorly soluble	No	Yes	-6.23 cm/s	Yes; 0 violation	0.55	0 alert	1 alert: mannich_A	4.33
**C5**	MolPort-000-451-711	7	1	66.15	4.65	141.75	High	-6.13 Poorly soluble	Yes	Yes	-5.46cm/s	Yes; 0 violation	0.55	0 alert	1 alert: mannich_A	4.57
**C6**	MolPort-000-451-749	7	1	89.21	2.86	111.93	High	-3.09 Poorly soluble	No	Yes	-7.69 cm/s	Yes; 0 violation	0.55	0 alert	0 alert	3.71
**C7**	MolPort-002-611-137	5	0	68.46	3.22	99.43	High	-4.33 Poorly soluble	No	Yes	-5.81 cm/s	Yes; 0 violation	0.55	0 alert	0 alert	3.65
**C8**	MolPort-002-619-190	3	0	95.45	6.74	139.5	Low	-7.48 Poorly soluble	No	Yes	-3.28 cm/s	Yes; 1 violation: MLOGP>4.15	0.55	0 alert	0 alert	4.17
**C9**	MolPort-002-608-446	3	0	95.45	6.47	134.69	Low	-7.17 Poorly soluble	No	Yes	-3.53 cm/s	Yes; 1 violation: MLOGP>4.15	0.55	0 alert	0 alert	4.04
**C10**	MolPort-000-451-699	6	1	75.80	4.47	132.78	High	-5.87 Moderately soluble	No	Yes	-5.36 cm/s	Yes; 0 violation	0.55	0 alert	1 alert: mannich_A	4.29

Table S2: The prediction of ADME parameters, pharmacokinetic properties, druglike nature, and medicinal chemistry friendliness of top 10 lead compounds predicted potent inhibitors against PCNA target.

Serial No.	Compounds (MolPort IDs)	H-Bond Acceptors	H-Bond Donors	TPSA (Å2)	Consensus Log $P_{o/w}$	Molar Refractivity	GI Absorption	Log S	BBB Permeant	P-gp Substrate	Log Kp (skin permeation) cm/s	Drug likeness based on Lipinski rule	Bioavailability Score	PAINS (alert)	Brenk (alert)	Synthetic accessibility
**C1**	MolPort-001-741-093	10	3	159.05	-0.03	101.59	Low	-2.30 soluble	No	No	-9.07 cm/s	Yes; 0 violation	0.55	0 alert	1 alert: cumarine	5.38
**C2**	MolPort-000-700-443	6	3	107.02	0.66	83.82	High	-4.61 Moderately soluble	No	No	-6.11 cm/s	Yes; 0 violation	0.55	0 alert	0 alert	3.33
**C3**	MolPort-047-116-128	10	5	194.76	-1.75	78.81	Low	-0.81 Very soluble	No	No	-9.40 cm/s	Yes; 1 violation: NorO>10	0.55	0 alert	3 alerts: aldehyde, nitro_group, oxygen-nitrogen_single_bond	3.96
**C4**	MolPort-039-345-350	8	4	129.59	0.64	94.14	High	-2.62 soluble	No	No	-8.28 cm/s	Yes; 0 violation	0.55	0 alert	2 alerts: cumarine, polycyclic_aromatic_hydrocarbon_3	4.90
**C5**	MolPort-002-525-976	11	8	189.53	-3.64	68.16	High	1.44 highly soluble	No	Yes	-11.92 cm/s	No; 2 violations: NorO>10, NHorOH>5	0.17	0 alert	0 alert	5.35
**C6**	MolPort-046-836-802	13	8	215.83	-3.94	83.83	Low	1.82 highly soluble	No	Yes	-13.00 cm/s	No; 2 violations: NorO>10, NHorOH>5	0.17	0 alert	1 alert: aldehyde	5.68
**C7**	MolPort-020-232-354	5	3	87.14	2.02	107.97	High	-3.03 soluble	No	Yes	-7.57 cm/s	Yes; 0 violation	0.55	0 alert	0 alert	3.37

**C8**	MolPort-020-232-872	5	4	99.17	0.77	89.60	High	-1.94 Very soluble	No	Yes	-8.03 cm/s	Yes; 0 violation	0.55	0 alert	0 alert	2.84
**C9**	MolPort-004-860-220	6	2	81.08	3.08	123.15	High	-5.04 moderately soluble	No	No	-6.10 cm/s	Yes; 0 violation	0.55	0 alert	0 alert	3.58
**C10**	MolPort-044-727-363	10	6	173.98	0.18	102.91	Low	-3.18 soluble	No	Yes	-8.29 cm/s	Yes; 1 violation: NHorOH>5	0.55	1 alert: quinone_A	0 alert	5.01
